# Evaluation of multisectoral interprofessional collaboration for non-communicable disease management within a municipal setting: a mixed methods study

**DOI:** 10.1186/s12875-025-02937-4

**Published:** 2025-08-05

**Authors:** Salomey Asaah Denkyira, Kwaku Gyamfi Oppong, Daniel Boateng, Joseph Attakorah, Kwame Ohene Buabeng

**Affiliations:** 1 Faculty of Pharmacy and Pharmaceutical Sciences, Department of Pharmacy Practice, Kwame Nkrumah University of Science and Technology, Kumasi, Ghana; 2 University of Ghana Medical Centre, Pharmacy Directorate, Accra, Ghana; 3 Komfo Anokye Teaching Hospital, Child Health Directorate, Kumasi, Ghana; 4 School of Public Health, Department of Epidemiology, Kwame Nkrumah University of Science and Technology, Kumasi, Ghana; 5 School of Pharmacy, Department of Pharmacy Practice, University of Health and Allied Sciences, Ho, Ghana

**Keywords:** Interprofessional collaboration, Non-communicable diseases, Community pharmacy, Healthcare professionals, Municipality

## Abstract

**Background:**

Strengthening multisectoral collaboration is essential for NCD prevention and control. In Ghana, the healthcare system often experiences inefficiencies due to fragmented care pathways and limited patient engagement. In this study, we assessed the collaboration and level of satisfaction on collaboration between healthcare professionals (HCPs) in hospital settings and Community pharmacists (CPs) in community pharmacies in the management of Diabetic/hypertensive (DM/HPT) patients in Ghana.

**Method:**

This was a cross-sectional study conducted in the Oforikrom municipality in Kumasi Metropolis. A mixed methods approach was used. Participants included all CPs and HCPs practicing in the municipality, as well as DM/HPT patients receiving care at the study site. Quantitative data was collected through online questionnaires. To gather qualitative insights, semi-structured interviews were further conducted. The interviews were audio-recorded and transcribed, and key themes were extracted.

**Results:**

A total of 170 participants (HCPs (*n* = 30), CPs (*n* = 39), and DM/HPT patients (*n* = 101)) completed the survey. Subsequently, seven (7) CPs and five (5) HCPs also participated in interviews. The study showed that CPs, HCPs and DM/HPT patients collaborated through practices such as sending referrals & making recommendations for therapy, follow-ups on patients’ therapy and outcomes assessment, providing feedback, adherence support and reminders. The quantitative data suggested a potential communication gap in the referral process between CPs and HCPs. The qualitative data showed that, despite both CPs and HCPs acknowledging the benefits of collaborative practice, it is currently limited. Furthermore, the study revealed varied levels of satisfaction on collaboration for NCD management. An equal proportion (40%) of both CPs and HCPs expressed satisfaction with their collaborative practices with each other, and 25% from each group reported dissatisfaction. The remaining participants were neither satisfied nor dissatisfied. The majority (78%) of CPs and an appreciable proportion of patients (34%) were satisfied with their collaborative relationship for patient care. However, a significant proportion (60%) of patients were dissatisfied with the overall collaboration between HCPs and CPs.

**Conclusion:**

The study highlights gaps in collaborative practice between healthcare providers (CPs, HCPs) and NCD patients, notably revealing patient dissatisfaction with the overall inter-professional collaboration, indicating a need for improved communication, bi-directional referral systems, and teamwork to optimize care and improve health outcomes.

**Supplementary Information:**

The online version contains supplementary material available at 10.1186/s12875-025-02937-4.

## Background

Non-communicable diseases (NCDs) such as diabetes, and hypertension pose a significant burden on healthcare systems worldwide [1, 2]. Their prevalence is of particular concern in municipalities, where population density and limited resources can exacerbate challenges in managing these chronic conditions [3, 4]. According to the World Health Organization, globally, NCDs pose a major health threat, causing 41 million deaths annually, equivalent to 74% of all global deaths. These diseases also have a devastating impact on younger populations, with 17 million people dying prematurely (before age 70) [5, 6]. Alarmingly, 86% of these premature deaths occur in low- and middle-income countries [6]. In Ghana, NCDs account for an estimated 45% deaths and 21% of premature mortality [7].

In recent decades, NCDs have emerged as a major health concern in Ghana, significantly impacting morbidity and mortality [8]. While comprehensive population-level data on NCDs in Ghana remains limited, available evidence from research and surveys—including findings from the Ministry of Health’s Global Burden of Disease study—strongly suggests a substantial rise in NCDs and their risk factors throughout Ghana [8, 9].

A key objective of the Ghana National Policy on NCDs is to foster multi-sectoral collaboration to effectively prevent and control these diseases [8]. Leveraging the private sector, particularly CPs, is crucial for achieving this goal. However, inefficiencies within the healthcare system in Ghana, including fragmented care pathways and limited patient engagement, hinder optimal integration of CPs [10]. Barriers to this integration include insufficient pharmacist input in healthcare pathways, strained relationships between pharmacists, physicians, and other healthcare workers, limited access to patient information, logistical constraints (time and distance), and inadequate remuneration [11, 12]. The overarching challenge lies in the lack of inter-professional collaboration and the suboptimal functioning of the private primary care network in managing NCDs [8, 9].

Extensive research has criticized the model of medical practice in which physicians often act as sole providers, assuming responsibility for diagnosis, dispensing, patient education, and counseling, sometimes with limited support from inadequately trained medical assistants or nurses [13]. This practice raises concerns, particularly given the lack of formal pharmaceutical training for physicians compared to pharmacists. Furthermore, instances of professional misconduct have also been documented among CPs, such as managing complex conditions without appropriate referrals, dispensing medications without valid prescriptions, and unauthorized medication adjustments [14, 15]. These cases overshadow the potential benefits of interprofessional collaboration. NCDs are complex and require a multidisciplinary approach. Interprofessional teams, comprising physicians, pharmacists, and other healthcare professionals, can provide more comprehensive patient care by leveraging the unique expertise of each member [16].

Research consistently demonstrates the underutilized potential of CPs in effectively managing NCDs [10]. Numerous studies have shown that pharmacist-led interventions significantly improve medication adherence, a critical factor in NCD control [17, 18]. Moreover, collaborative efforts between CPs and healthcare facilities foster a more comprehensive and integrated approach to NCD care [19–22]. Enhanced communication and information sharing between these professionals can lead to improved patient outcomes, reduced healthcare utilization, and lower overall costs [23–25].

A growing consensus among scholars, professional organizations, and policymakers recognizes the need for CPs to play a more active role in ensuring the safe, effective, and efficient use of medications, particularly for individuals with NCDs [10]. This expanded role necessitates seamless collaboration with healthcare facilities, including hospitals and clinics, to ensure continuity of care and facilitate timely interventions [26]. However, the success of this model hinges on empowering patients [27]. By actively engaging patients in their NCD management journey and providing them with the necessary knowledge and tools, we can transition towards a more patient-centered approach to healthcare.

Studies by Smilski & Parrott (2019) emphasize the importance of patient engagement and shared decision-making in successful NCD management. Empowering patients through education and providing them with the tools to manage their conditions actively will help lead to better compliance with treatment and improved outcomes [28]. A collaborative model that integrates patient perspectives and preferences can lead to improved adherence and overall well-being [19].

Municipal settings provide a localized approach for effective NCD management. This approach enables efficient resource allocation and the development of tailored interventions that address the specific needs of the community. By fostering collaboration among CPs, healthcare facilities, and patients, municipalities can leverage existing resources to create a more comprehensive NCD management system. This study aims to evaluate the current collaboration between these key stakeholders in the management of diabetes and hypertension (DM/HPT) within the Oforikrom municipality. The findings of this study will inform the development of a more streamlined, patient-centered, and efficient approach to NCD management within the municipality.

## Methods

### Study design

This study employed a mixed-methods design, combining quantitative and qualitative approaches. Data collection occurred between May and August 2023. Quantitative data was collected via online questionnaires administered to various participant groups by trained research assistants. These questionnaires assessed sociodemographic characteristics, specific collaborative practices (including referrals, adherence support, and reminders/follow-ups), and participant satisfaction with collaboration (i.e. completely dissatisfied, dissatisfied, neither satisfied nor dissatisfied, satisfied, very satisfied). Concurrently, qualitative data was gathered through semi-structured interviews conducted using an interview guide. Interviews were audio-recorded and subsequently transcribed. The interview guide explored collaborative working relationships between CPs and physicians, and the existence of referral systems.

### Study setting

The research took place in Oforikrom, an urban municipality in the Ashanti region of Ghana. This region boasts of a population of roughly 213,126 with a relatively even gender split. Notably, the majority (72%) fall within the working-age bracket of 15–64 years [29]. Oforikrom’s healthcare system is a mix of public and private providers, with 13 private facilities, 2 government facilities, and 1 quasi-government facility. Additionally, with the presence of 43 CPs it indicates a strong network of medication access points [30].

### Study population

It involved CPs, HCPs in Hospitals (Government, Private and Quasi) in the municipality and diabetes and hypertension patients within the Oforikrom municipality. A total of 170 participants (101 DM/HPT patients, 30 HCPs and 39 CPs) completed the survey for the quantitative method and seven (7) CPs and five (5) HCPs participated for the qualitative method.

### Inclusion criteria

The study included all CPs and HCPs working within the municipality, along with all DM/HPT patients accessing healthcare services at the study site.

### Exclusion criteria

Participants excluded from this study included those who did not provide informed consent, hospitalized diabetes or hypertension (DM/HPT), and hospital pharmacists. This study specifically focused on CPs within the private sector.

### Sampling

For the quantitative phase, all forty-three (43) CPs within the municipality were surveyed, out of which thirty-nine (39) participated. A convenience sample of thirty [31] healthcare professionals (HCPs) was recruited. To identify eligible DM/HPT patients, a two-pronged approach was employed: (1) requesting CPs to identify patients within their clientele, and (2) utilizing a snowball sampling technique to expand the participant pool. This resulted in a sample of one hundred and one (101) DM/HPT patients. For the qualitative phase, participant recruitment utilizes a convenience sampling method. A total of 12 participants: CPs (*n* = 7) and physicians (*n* = 5) consented.

### Data collection & analysis

Quantitative data was collected through online questionnaires (Supplementary Appendix I) administered to all participant groups by three trained research assistants (S. A.F, B.M.G and A.N.A.T, see acknowledgements). Data was descriptively analyzed using Stata^®^ 17 software, with continuous variables reported as mean ± SD or median. Categorical variables were reported as percentages and proportions. Qualitative data was gathered through 20-to-30-minute interviews conducted by two trained qualitative interviewers (P.M.N and PA, see acknowledgements). The interview guide generally focused on: collaborative working relationships (CPs, physicians) and the existence of referral systems (CPs, physicians). All interviews were transcribed and analyzed by an independent researcher using NVivo version 14 software. Transcripts were initially reviewed to gain an overall understanding of the phenomenon. Key concepts were identified and coded, leading to the development of evolving themes and subthemes. Final themes were determined through consensus discussions within the research team. Both the online questionnaire and interview guide for each participant group were developed based on a review of relevant literature [19, 31–33]. (supplementary appendix II) and was piloted with 10 CPs.

### Outcome measures

Descriptive statistics on collaborative practices such as referrals, follow-ups, feedback, recommendations, adherence support, reminders and satisfaction on collaboration were assessed for the quantitative data. Qualitative data analysis focused on identifying key themes related to collaborative working relationships between CPs and HCPs, as well as the existence and effectiveness of referral systems.

## Results

Demographics for each of the participant groups are presented in Tables 1 and 2. Most of the pharmacies (*n* = 35, 89.7%) were retail pharmacies. The mean number of years of operation of these pharmacies was 10.4 *±* 8.5 years (range 2 to 42 years). Only (*n* = 5,12.8%) of the pharmacies had more than two working pharmacists. Most of the pharmacists (*n* = 18, 46%) attend to at most ten DM/HPT patients in a month. Of the 16 healthcare facilities identified in the study site, a total of 30 healthcare professionals participated in the study. Almost half of the healthcare professionals (*n* = 13, 43.3%) were physicians and the majority (*n* = 19, 63%) were from the government hospitals (Table 1).

**Table 1:** Characteristics of the CPs and healthcare professionals in hospital settings

Variable	Frequency (*n* = 39)	Percentage (%)
Type of pharmacy		
Retail	35	89.7
Wholesale	0	0.0
Both	4	10.3
Number of years of operation		
* ≤* 5	19	48.7
6–10	7	18.0
> 10	13	33.3
Number of pharmacists working in the pharmacy		
* ≤* 2	34	87.2
> 2	5	12.8
Number of support staff working in the pharmacy		
≤ 2	21	53.8
> 2	18	46.2
Number of hypertensive/diabetic patients seen in a month		
* ≤* 10	18	46.2
11–20	10	25.6
21–30	8	20.5
> 30	3	7.7
Healthcare Professionals	Frequency (*n* = 30)	Percentage (%)
In Hospitals		
Physician	13	43.3
Midwife	8	26.7
Nurse	9	30.0
Place of work		
Government hospitals	19	63.0
Private hospitals	8	27.0
Quasi hospitals	3	10.0

A total of 101 patients were involved in this study, with a slightly higher proportion of females (*n* = 54, 53.5%) compared to males (*n* = 47, 46.5%). The mean age of participants was 63.7 *±* 13.0 years (range 18 to 97 years) with majority of participants aged 61–70 years (39.6%), followed by those aged 51–60 years (26.7%). A significant proportion (*n* = 59, 58.4%) had tertiary education, while (*n* = 56, 55.4%) were married. Hypertension was the most prevalent condition (*n* = 47, 46.5%), followed by those who had both hypertension and diabetes (*n* = 44, 43.6%). A notable percentage (*n* = 84, 83.2%) of participants had a family history of hypertension (Table 2).

**Table 2:** Sociodemographic characteristics of patients

Variable	Number	Percentage
Sex		
Male	47	46.5
Female	54	53.5
Age (years)		
< 40	6	5.9
41–50	5	5.0
51–60	27	26.7
61–70	40	39.6
71–80	16	15.9
> 80	7	6.9
Marital status		
Never married	9	8.9
Married	56	55.4
Separated/Divorced	13	12.9
Widowed	23	22.8
Level of education		
No formal education	9	8.9
Primary	15	14.9
Secondary	18	17.8
Tertiary	59	58.4
Participants’ condition		
Diabetes	10	9.9
Hypertension	47	46.5
Both	44	43.6
Number of condition years		
< 5	49	48.5
6–10	33	32.7
11–15	9	8.9
> 16	10	9.9
Family history		
Hypertension	84	83.2
Diabetes	52	51.5
Heart attack	2	2.0
Stroke	6	5.9

### Collaboration between CPs and DM/HPT patients

As shown in Table 3, CPs provide various collaborative services to patients in the community. Nearly half (*n* = 16, 41%) of the CPs reported providing both follow up after patients’ visits and adherence support for patients. Two-thirds of the CPs mentioned that they sometimes received feedback from diabetic/hypertensive patients on their general wellbeing. Although some (*n* = 20, 51.2%) indicated they received feedback from patients on drug-related concerns. For cases beyond the scope of CPs, majority (*n* = 31, 71.9%) of the CPs made a referral/recommendation to a hospital. Conversely, with patients’ acknowledgement of CP roles, the majority of patients responded never to receive adherence support (*n* = 54. 53.5%) nor follow-ups/reminders (*n* = 73, 72.2%) from CPs after patients’ visit. Almost a quarter of the DM/HPT patients (*n* = 23, 22.8%) also indicated that they receive information/counseling from pharmacists on their condition (Table 4).

**Table 3:** Collaborative practices undertaken by CPs

Collaborative practices	Never *N* (%)	Rarely *N* (%)	Sometimes *N* (%)	Every time *N* (%)
Adherence support for patients	5 (12.8)	10 (25.7)	16 (41.0)	8 (20.5)
Follow up after patients’ visits	7 (17.9)	9 (23.2)	16 (41.0)	7 (17.9)
Elicit patient feedback on well-being	2 (5.1)	4 (10.3)	26 (66.7)	7 (17.9)
Elicit patient feedback on drug related concerns	3 (7.7)	9 (23.1)	20 (51.2)	7 (17.9)
Hospital referrals for conditions exceeding the scope of community pharmacy services	0 (0.0)	1 (2.6)	7 (17.9)	31 (79.5)

**Table 4:** Patients’ acknowledgment of CPs collaboration

Collaborative practices	Never *N* (%)	Rarely *N* (%)	Sometimes *N* (%)	Every time *N* (%)
Provision of information/counseling by pharmacist on your condition.	35 (34.6)	15 (14.9)	28 (27.7)	23 (22.8)
Adherence support from pharmacist	54 (53.5)	8 (7.9)	19 (18.8)	20 (19.8)
Pharmacist sends reminders or follow ups for refills	73 (72.2)	12 (11.9)	12 (11.9)	4 (4.0)
Frequency of sending feedback to community pharmacy	69 (68.3)	11 (10.9)	16 (15.8)	3 (5.0)

### Collaboration between CPs and HCPs

Patient referrals constituted the primary form of collaboration between CPs and HCPs. The majority of CPs (*n* = 26, 64.7%) send 5 referrals in a month at most to healthcare facilities. Largely, (*n* = 20, 62.5%) referrals are sent via written notes. More than half (*n* = 26, 66%) indicated they referred to these sites due to the proximity of these health facilities to the pharmacy (Table 5).

**Table 5:** CP-provided collaborative services to HCPs

Variable	Frequency (%)
Number of referrals in a month	
* ≤* 5	26 (64.7)
6–10	7 (17.9)
> 10	6 (15.4)
Location of referral hospital	
Within the municipality	29 (74.4)
Outside the municipality	3 (7.7)
Sometimes within sometimes outside	7 (17.9)
Reasons for referral	
Proximity to the pharmacy	26 (66.7)
Good provision of healthcare services	25 (64.1)
Presence of a specialized professional	16 (41.0)
Affordability of healthcare	18 (46.2)
Good working relationship	10 (25.6)
Personal/Friendly relationship	6 (15.4)
Proximity to patients	4 (10.3)
Means of referral	
Calls via telephone	3 (9.4)
By written note	20 (62.5)
In-person	15 (46.8)

While a major proportion (79.5%, *n* = 31) of CPs initiate referrals (Table 3), feedback on these referrals are limited. Some HCPs (56.7%, *n* = 17) acknowledged receiving referrals, others (23%, *n* = 7) reported receiving none. More than 40% of HCPs indicated they never send feedback to CPs after receiving referred cases (Table 6).

**Table 6:** HCPs’ collaboration with cps: acknowledgment and practices

Statement	Never *N* (%)	Rarely *N* (%)	Sometimes *N* (%)	Every time *N* (%)
Referral/recommendation from CPs	3(10.0)	7 (23.3)	17 (56.7)	3 (10.0)
Frequency of sending feedback to CPs about referred cases	12(41.4)	9 (31.0)	7 (24.2)	1 (3.4)
Follow up on diabetic/hypertensive patients seen at the facility to CPs	17(58.6)	4 (13.8)	7 (24.2)	1 (3.4)

### Satisfaction with collaboration

A multi-group analysis of satisfaction with collaboration in NCD management revealed varied perceptions. CPs and HCPs exhibited similar levels of satisfaction (approximately 40%) and dissatisfaction (approximately 25%) with their collaborative efforts. However, a more positive dynamic emerged between pharmacists and DM/HPT patients, with a high satisfaction rate among pharmacists (78%) and a moderate satisfaction rate among patients (34%) on their collaboration towards each other. Conversely, a significant number of DM/HPT patients (60%) expressed dissatisfaction with the level of collaboration between HCPs and CPs in managing their conditions as shown in (Table 7).

**Table 7:** Satisfaction with collaboration

Study groups	Completely dissatisfied %	Dissatisfied %	Neither satisfied nor dissatisfied %	Satisfied %	Very satisfied %
CPs satisfaction with HCPs’ collaboration	5	26	26	38	5
HCPs satisfaction with CPs’ collaboration	3	27	23	40	7
CPs satisfaction with patients’ collaboration	**-**	**-**	22	78	**-**
Patients’ satisfaction with CPs’ collaboration	15	25	26	34	-
Patients’ satisfaction with collaboration between CPs and HCPs	-	60	26	14	**-**

### Qualitative analysis

The majority of the HCPs (7) were pharmacists, and 5 were physicians. Five of the pharmacists have been working as CPss for two years and more. All the five medical practitioners have been practicing for over a period of 2 years.

**Table:** 

Themes	Subthemes
Collaboration between Physicians and CPs	1. CPs and Their Relationship with physicians 2. Referral System Between CPs and physician

### Collaboration between Physicians and CPs

This theme describes the relationship between CPs and the physicians which is critical in achieving good health outcome of the patient. Patient care is a team approach rather than individualistic care. Achieving the best care for clients depends on how cooperative physician and CPs relate. All participants described how best they relate with each other and sometimes what strains their relationship.

#### *Subtheme 1: CPs and their relationship with physicians*

Collaboration between health workers allows the sharing of important information about clients. Collaborating with a physician as a CPs will help him to catch up with patient medical history which will help in terms of drug intervention. All CPs revealed that there is no collaboration with physicians. However, one physician responded that there is an actual pharmacist they collaborate with and refer their client there to purchase medication. All CPs responded that the lack of collaboration can be linked to uncooperativeness of physicians One CPs strongly believed that the lack of collaboration is because of unfamiliarity of the junior medical officer with the health system.

*“You see as medical doctors “you don’t touch me” “I am a doctor*,* do your work and let me do my work”. The problems come from the young doctors who are coming up recently” (Participant 8*,* a CPs).*

All study participants testified that there is a huge benefit in collaborating with each other. One medical practitioner said that collaborating with CPs will help to decongest the OPD and helps reduce the constant visit of clients to the health facility. Another medical practitioner responded affirmative to the benefit of collaboration as he expressed;

*“With CPs*,* any benefit I can think of this*,* it releases stress on the patient. Like if you have a patient who is stable for month*,* the patient can go to the nearby community pharmacy where they can pick some of their medications. And then occasional every two or three months*,* the patient can pass by the hospital for checkups. That one can reduce the patient stress. It also reduces the time the patient will spend in the facility” (Participant 5*,* a medical practitioner).*

When asked about the benefit of collaborating with the health facility, one CPs responded;

“*Yes*,* in terms of the fact that this job is patient centered so a systematic collaboration basically between medical doctors and pharmacy would be perfect in order to help patient with their drugs*,* monitoring to see whether it will help the patient or change it”. (Participant 6*,* a CPs***).**

#### *Subtheme 2: Referral system between community pharmacies and health facilities*

When asked about any referral system put in place when referring a client to the health facility or the community pharmacy, none of the health workers responded to have a referral system when referring to the community pharmacy and only one of the CPs responded that he has a referral and feedback system for his referrals. All CPs responded that if they assess any client and realize the person needs referral, they immediately refer the person to the nearest health facility. The only nurse who partook in the interview responded that CPs always calls when referring a case to the facility. One of the CPs expressed his concern on the lack of referral system which strains their communication with the physicians as;

*“On referral*,* it becomes very very difficult. Just like in the hospital setting you have a referral form*,* anytime you have a client*,* but the case is beyond you*,* you can fill the forms and just refer to the facility. But usually the protocol is*,* before you refer you call the facility to make sure there is space and another healthcare professional who is ready to receive the patient before you transfer or refer. We need a referral system linking us and the facility just like facility to facility” (Participant 2*,* a CPs).*

## Discussion

The goal of the study was to assess collaboration practices between CPs, HCPs and patients in the management of DM/HPT. Although, the findings identified existing collaborative practices, they also revealed significant challenges that impede effective collaboration and ultimately, patient care in Ghana. This was similar to previous studies which emphasizes the need to develop strategies to improve collaborative relationships between healthcare professionals and pharmacists [31–34].

The study revealed the use of some collaborative practices like referrals, follow-ups, feedback, recommendations, adherence support and reminders. These collaborative practices have been seen in previous studies in assessing collaboration between healthcare professionals [26, 35]. The quantitative data exposed a potential communication gap in the referral process. A high proportion of pharmacists reported initiating referrals; however, a substantial number of HCPs did not acknowledge receiving them. This lack of a closed-loop communication system, where referrals lack confirmation and feedback, hinders collaborative patient management. Hence, the implementation of a bi-directional referral system will go a long way to ensure effective collaboration between healthcare providers and CPs and this will not only ensure continuity of care but also improve patients’ health outcomes.

The study also identified a disconnect between pharmacist-reported services and patient experiences. Pharmacists reported providing adherence support and medication reminders, yet a significant portion of patients perceive not receiving these services. This discrepancy necessitates further investigation into communication barriers and potential issues with patient engagement strategies. Are language barriers hindering communication? Are medication reminders poorly designed or delivered at inconvenient times? Understanding the root causes of this gap is essential for developing targeted interventions. Pharmacists may need to improve communication techniques or tailor their strategies to better suit individual patient’s needs and preferences.

From the qualitative data,

despite acknowledging the benefits of collaboration from both participant groups (i.e. CPs and HCPs), they also highlighted limitations in its current implementation. Pharmacists attributed this to limited cooperation, particularly from junior HCPs unfamiliar with established referral systems. Interestingly, a unidirectional referral flow emerged again, with pharmacists referring patients to HCPs but no formal system for HCPs to direct patients to pharmacists. This one-way flow suggests a missed opportunity to leverage the expertise of CPs. This result has been evident in previous literature where integration of CPs has been faced with many challenges [10, 36].

Furthermore, the study documented varied levels of satisfaction with collaboration among participant groups. While CPs and HCPs displayed approximately similar levels of satisfaction and dissatisfaction with their collaborative efforts, a significant number of patients expressed dissatisfaction on collaboration between CPs and HCPs. This highlights the need for interventions that bridge the gap between perceived benefits and the current reality of collaboration in NCD care. If patients are unaware of or unsatisfied with the collaborative efforts between CPs and HCPs, their treatment adherence and overall health outcomes may suffer. Addressing these concerns is essential for ensuring a patient-centered approach to NCD management.

These findings call for strategic actions to improve collaboration across all stakeholders by implementing robust communication channels, potentially through electronic health records or a common dedicated platform to ensure complete referral loops and timely feedback. Also, establishing clear and standardized referral protocols, including bi-directional referrals between CPs and HCPs, can streamline patient care pathways. Furthermore, developing and implementing educational programs for both CPs and HCPs focusing on interprofessional collaboration, referral systems, and effective communication can improve teamwork and understanding. Finally, investigating and addressing communication barriers that hinder patient awareness of pharmacist-provided services is crucial for maximizing patient engagement and adherence to treatment plans. This may involve developing culturally sensitive communication materials, providing medication reminders in preferred formats (text messages, phone calls), or offering medication synchronization services to simplify medication regimens.

### Limitations of the study

The qualitative data was collected generally through open face-to-face interviews, which may have introduced bias as the interview setting itself may have influenced their responses. Furthermore, the use of purposive and snowball sampling strategies may have introduced selection bias, limiting the generalizability of the findings to the specific context of the Oforikrom municipality. Also, pharmacists may have been more likely to select patients satisfied with their collaborative practices, although the diversity of responses suggests this bias was minimal. Finally, as with all studies, the findings are representative of those who participated and may not fully reflect the experiences of the entire population.

## Conclusions

The study revealed existing collaborative practices like referrals and follow-ups on therapy to monitor patient outcomes. The quantitative data suggested a potential communication gap in the referral process. Specifically, there was a discrepancy between the rate of CPs-initiated referrals and the proportion of HCPs in hospitals acknowledging receipt of these referrals. The study further identified a potential disconnect between pharmacist-reported services and patient experiences. The qualitative data, however, presented a more nuanced picture. Despite both CPs and HCPs acknowledging the benefits of collaboration, the study revealed its currently limited. Finally, the study documented varied satisfaction rate with collaboration among the participant groups in NCD management. These major findings underscore the need for strategic interventions to enhance collaboration across all stakeholders involved in NCD care within health systems in communities. Efforts should focus on improving communication channels, establishing bi-directional referral systems, and fostering a culture of teamwork. By addressing these areas for improvement, healthcare systems can create a more collaborative and patient-centered environment, ultimately leading to better outcomes for patients with NCDs.

## Recommendations

In future studies, consider using a more diverse sampling strategy, such as random sampling or stratified sampling, to increase the representativeness of the study population. This would improve the generalizability of the findings to a wider population. Furthermore, utilize multiple recruitment strategies to minimize selection bias. This could include recruiting participants through community health centers, public health campaigns, and online platforms.

## Supplementary Information

Supplementary Material 1.

## Data Availability

The author confirms that all data generated or analysed during this study are included in this published article. example from https://doi.org/10.1016/j.healthpol.2015.02.007.

## Abbreviations

CPs: CPs
HCPs: Healthcare Professionals
NCDs: Non communicable diseases
DM/HPT: Diabetes/Hypertension
