# Supplementary material for: Evaluation of multisectoral interprofessional collaboration for non-communicable disease management within a municipal setting: a mixed methods study
Source: BMC Prim Care. 2025 Aug 5;26:245. doi: 10.1186/s12875-025-02937-4 (PMC12326643; doi:10.1186/s12875-025-02937-4)
Supplement: Supplementary file 1 — Supplementary Material 1. [file 12875_2025_2937_MOESM1_ESM.docx]

**APPENDIX I**

**Community pharmacists**

1. Name of the Pharmacy?
2. How long has the pharmacy been in operation?
3. Number of pharmacists working in this facility? (In figures; 1,2,3 etc.)
4. How many pharmacy support staff are working in this facility?  (In figures; 1,2,3 etc.)

- ***Pharmacist/Patient collaboration (The Pharmacist activities rendered to the patient)***

1. On average, how many Diabetes and Hypertension patients do you see in a month? (In figures; 1,2,3 etc.)
2. Do you provide adherence support for Diabetes and Hypertension patients?

Never, Rarely, Sometimes, Every time

1. Do you follow up after patient visits?

Never, Rarely, Sometimes, Every time

1. Do you usually send reminders to Diabetes and Hypertension patients on their re-fills? Y/N
2. If yes, how are these reminders sent?

In- person, Calls via telephone, Text messages, By mail, written notes

1. Do you get feedback from Diabetes and Hypertension patients on their general wellbeing and study progress on the management of their condition?

Never, Rarely, Sometimes, Every time

1. What is your level of satisfaction on the collaboration with Patients with DM/HTN on the management of their condition?

completely dissatisfied, dissatisfied, neither satisfied nor dissatisfied, satisfied, very satisfied

- ***Pharmacist/healthcare professionals’ collaboration (Pharmacist activities directed towards the HCP)***

1. In the identification of medical and drug related problems beyond your scope, do you subsequently make a referral/recommendation to the hospital?
2. Are the referrals/recommendations to hospitals within the municipality? Y/N
3. What are some specific reasons referring these Diabetes and Hypertension patients to this specific health facility?

Close proximity to pharmacy, Good provision of health care services, Presence of a specialized healthcare personnel, Affordability of healthcare, Good working relationships, Personal/Friendly relationships with healthcare personnels there

1. By what means are the recommendations/referrals made? Calls, written notes, in-person
2. On average how many referrals/recommendations do you make in a month to the hospitals?  (In figures; 1,2,3 etc.)
3. Do you get feedback from healthcare personnel in the hospital on referrals/recommendations made?

Never, Rarely, Sometimes, Every time

1. Do you find these feedbacks from healthcare personnel on referred Diabetes and Hypertension patients useful? Y/N
2. What is your level of satisfaction on the collaboration with healthcare personnel in hospitals on the management of Patients with DM/HTN?

completely dissatisfied, dissatisfied, neither satisfied nor dissatisfied, satisfied, very satisfied.

**For healthcare professionals**

1. What is the name of the facility in which you work?
2. What level of healthcare system does it fall?

Health centres, CHPS compound, Clinics, District hospitals, regional hospitals, Tertiary hospitals

1. What is your profession at this facility?

Physician, Physician assistant, Midwives, Nurse

- ***Healthcare professionals/Pharmacists’ collaboration (HCPs-Acknowledged pharmacist activities)***

1. Do you often get referrals/recommendations from community pharmacists on diabetes and hypertension cases? Never, Rarely, Sometimes, Every time
2. Are the referrals/recommendations from community pharmacists within the municipality? Y/N
3. On average in a month, how many of these referrals/recommendations do you receive? (In figures.1,2,3..)

- ***Healthcare professionals/Pharmacists’ collaboration (HCPs activities)***

1. Do you also send feedback to the community pharmacists on referrals or recommendations made by them? Never, Rarely, Sometimes, Every time
2. Do you send follow-ups of diabetes and hypertension cases seen at the hospital to community pharmacists for continuum of care? Never, Rarely, Sometimes, Every time
3. What is your level of satisfaction on the collaboration with community pharmacists on the management of Patients with DM/HTN?

completely dissatisfied, dissatisfied, neither satisfied nor dissatisfied, satisfied, very satisfied.

**For Patients with DM/HTN**

- **Sociodemographics**

1. Sex? Male, Female
2. Age?
3. What is your occupation?
4. Marital status

Single, married separated/divorced widowed.

1. What is your highest level of education?

Uneducated, Primary/Jhs Senior High, Training college/university

1. What conditions do you have?

Diabetes Hypertension Both

1. How long have you had the condition? (In figures; 1,2,3 etc.)
2. Do you have any family history of the following?

Diabetes, Hypertension, Stroke, Heart attack, others

**Patient-pharmacist relationship**

1. Does the pharmacist provide you with education and counselling on your condition and medication? Never, Rarely, Sometimes, Every time
2. Do they provide you with adherence support?

Never, Rarely, Sometimes, Every time

1. Do they usually send reminders/follow ups your re-fills?

Never, Rarely, Sometimes, Every time

1. Do you also send feedback to community pharmacists on your general wellbeing and study progress on the management of your condition? Y/N
2. What is your level of satisfaction on the collaboration with pharmacists on the management of your DM/HPT?

Completely dissatisfied, dissatisfied, neither satisfied nor dissatisfied, satisfied, very satisfied.

1. What is your level of satisfaction on th collaboration between healthcare facilities and community pharmacists on the management of your condition? Completely dissatisfied, dissatisfied, neither satisfied nor dissatisfied, satisfied, very satisfied.

**APPENDIX II**

Interview Guide

Level of collaboration

1. Is there any collaboration between physicians and community pharmacists?
2. What are some collaborative practices in existence?
3. What are the benefits of collaborative practice between healthcare facilities, community pharmacists on management of DM/HPT?
